# Consumer insights into the at‐home liking of commercial beers: Integrating nonvolatile and volatile flavor chemometrics

**DOI:** 10.1002/fsn3.4066

**Published:** 2024-03-07

**Authors:** Perla A. Ramos‐Parra, Irma C. De Anda‐Lobo, Claudia Gonzalez Viejo, Raúl Villarreal‐Lara, Jorge Abraham Clorio‐Carillo, Luis Martín Marín‐Obispo, Diana Jessica Obispo‐Fortunato, Zamantha Escobedo‐Avellaneda, Sigfredo Fuentes, Esther Pérez‐Carillo, Carmen Hernandez‐Brenes

**Affiliations:** ^1^ Tecnologico de Monterrey Escuela de Ingeniería y Ciencias Monterrey NL México; ^2^ Digital Agriculture, Food and Wine Research Group, Faculty of Sciences The University of Melbourne Parkville Victoria Australia; ^3^ SensoLab Solutions Centro de Innovación y Transferencia Tecnológica (CIT2) Monterrey Mexico; ^4^ Tecnologico de Monterrey Institute for Obesity Research Monterrey NL México

**Keywords:** acceptability, beer, bitterness, chemometric, fermentation, style

## Abstract

Consumer acceptability of beers is influenced by product formulation and processing conditions, which impart unique sensory profiles. This study used multivariate techniques to evaluate at‐home consumer sensory acceptability of six commercial beers considering their style, fermentation type, and chemical composition. Samples included top‐fermented beers (American India Pale Ale and Stout) and bottom‐fermented beers (Pilsner, zero‐alcohol Pilsner, Vienna Lager, and Munich Dunkel). Beer consumers (*n* = 50) conducted sensory hedonic, check‐all‐that‐apply (CATA) and just‐about‐right (JAR) tests. Chemometric variables included iso‐alpha‐acids, hordenine, and volatile aromatic compounds, quantified by chromatographic methods, whereas bitterness units (IBU) were determined spectrophotometrically. Lager beers had higher acceptability than top‐fermented beer (*p* < .05) for all attributes. Light‐colored beers and medium‐height foams had the highest liking scores for visual sensory attributes. Higher concentrations of bitter‐tasting molecules, hordenine, and acidity decreased the liking scores of top‐fermented (Ale) beers, as a sensory penalty analysis suggested. In contrast, the most favored beers (Pilsners and Munich Dunkel) contained higher fusel alcohol esters linked to fruity aromatic notes. Although a low conversion rate of fatty acids into fruity esters was noted in nonalcoholic Pilsner, its overall liking score was not statistically different from the alcoholic version. However, consumers perceived the nonalcoholic Pilsner as less bitter than its alcoholic counterpart even when IBUs were nonsignificantly different. This study emphasized the significance of understanding beer chemometrics to comprehend consumer acceptability, highlighting the crucial role of bitter molecules. Hence, hordenine, acidity, and volatile contents provided additional and valuable insights into consumer preferences.

## Graphical abstract

Most liked beers (Pilsners and Munich Dunkel) contained higher fusel alcohol esters linked to fruity aromatic notes. Consumers penalized top fermentation beers characterized by higher concentrations of bitter molecules and hordenine. Light‐colored and medium‐height foam were the highest scored sensory visual attributes.

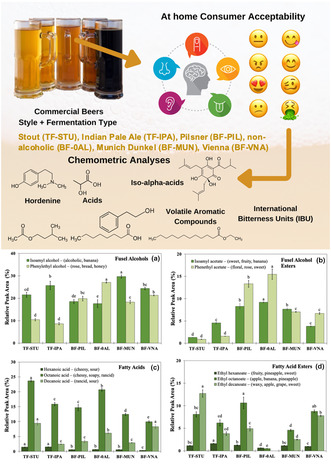

## 1 INTRODUCTION

Beer is the most widely sold alcoholic beverage in Mexico, which in 2020 ranked sixth in the world beer market (equivalent to 22.6 billion dollars). In 2020, approximately 8 million liters of beer were sold in Mexico (STATISTA, 2022); representing a research opportunity for understanding potential relationships between beer composition and consumer sensory preferences.

Barley (*Hordeum vulgare*) is the primary cereal in beer malt, while other grain adjuncts are used to enhance flavors or minerals (Parker, 2012; Serna‐Saldivar, 2016). Hordenine is a barley alkaloid that has gained attention due to its potential health benefits, and it also contributes with bitter flavor notes (Shahidi & Naczk, 2003; Sommer et al., 2019).

Another crucial raw material is hops (*Humulus lupulus*). Despite being added in small quantities, hops impact bitterness, hoppy flavor, provides antimicrobial properties, and aid in foam formation and stability (Parker, 2012; Serna‐Saldivar, 2016). Hops release α‐acids that are isomerized into iso‐α‐acids during wort boiling, which are highly bitter and soluble. Of the resulting isomers, *cis*‐isohumulone is the most bitter and *trans*‐isohumulone the least bitter. These molecules can produce fruity and hoppy scents and may deliver up to 85% of the bitterness in beers (Almaguer et al., 2014). Bitterness levels are known to markedly affect beer choices (Betancur et al., 2020).

Analyzing volatiles in beer is complex, since it contains more than 800 chemical compounds derived from its main ingredients (Olaniran et al., 2017). Many of these compounds are formed at different processing stages and can impact consumer preferences. Studies on sensory acceptability have consistently proven that beer bitterness levels are inversely related to overall acceptability (Hayward et al., 2019; Viejo et al., 2019). However, it has been shown that geographic locations affect the liking of darker and bitter beers (Van Doorn et al., 2019). Novel flavors may also affect the liking scores of samples tasted in the same sensory session. For instance Mexican consumers assigned significantly higher liking to fruity beers in contrast with commonly consumed top and bottom fermentation beers; the study included low bitterness Belgium specialty beers of spontaneous fermentation with cherry and raspberry notes (Gonzalez Viejo et al., 2019).

The present study was conducted during the pandemic confinement, a time in which 82% of beer was consumed at home (62.9 liters per capita in Mexico) (STATISTA, 2022). Therefore, this study aimed to assess at‐home consumer sensory acceptability of six commercial beers based on their style, fermentation type, and chemical composition using multivariate techniques. Chemometric variables included bitterness (IBU), iso‐α‐acids (IAA), hordenine, and volatile aromatic compounds used to integrate knowledge and build models with insights of consumer preferences for commercial beers.

## 2 MATERIALS AND METHODS

Commercially available top‐fermented beers (American India Pale Ale and Stout) and bottom‐fermented beers (Pilsner, zero‐alcohol Pilsner, Vienna Lager, and Munich Dunkel) were analyzed, including different beer styles defined by international beer standard associations. The selected styles were expected to result in different sensory profiles since they are characterized by the formulation and processing parameters described in Figure S1. The number of samples was limited due to the restriction of the maximum number feasible to assess in one sensory session with consumers. Within the four bottom fermentation samples, the Pilsner beer was contrasted in an alcoholic and nonalcoholic version. Sample characteristics and the alcohol content reported on the commercial label are summarized in Table 1.

**TABLE 1:** Sensory consumer results for six different beer brands commercialized in Mexico.

Attributes	TF‐STU	TF‐IPA	BF‐PIL	BF‐0AL	BF‐MUN	BF‐VNA
Foam Height	8.918 ± 0.447^ab^	8.846 ± 0.680^ab^	10.035 ± 0.482^a^	8.954 ± 0.503^ab^	10.146 ± 0.429^a^	7.897 ± 0.577^b^
Foam Stability	8.729 ± 0.418^a^	8.835 ± 0.556^a^	9.284 ± 0.501^a^	8.825 ± 0.460^a^	9.395 ± 0.505^a^	9.133 ± 0.542^a^
Visual Color	7.089 ± 0.652^b^	10.670 ± 0.416^a^	10.271 ± 0.418^a^	10.034 ± 0.434^a^	9.348 ± 0.496^a^	9.447 ± 0.489^a^
Clarity	6.020 ± 0.587^c^	10.461 ± 0.402^ab^	10.698 ± 0.433^a^	10.139 ± 0.418^ab^	9.220 ± 0.483^b^	9.593 ± 0.424^ab^
Aroma	8.026 ± 0.595^b^	9.695 ± 0.619^a^	9.738 ± 0.392^a^	9.169 ± 0.461^ab^	10.139 ± 0.459^a^	9.240 ± 0.485^ab^
Bitterness	5.671 ± 0.610^b^	6.028 ± 0.699^b^	9.119 ± 0.438^a^	8.795 ± 0.539^a^	8.056 ± 0.561^a^	9.144 ± 0.496^a^
Acidity	6.428 ± 0.518^c^	6.712 ± 0.616^bc^	8.599 ± 0.444^a^	8.038 ± 0.422^ab^	7.941 ± 0.504^ab^	8.534 ± 0.435^a^
Sweetness	5.950 ± 0.515^c^	6.654 ± 0.625^bc^	8.896 ± 0.499^a^	8.102 ± 0.466^ab^	7.824 ± 0.549^ab^	8.755 ± 0.456^a^
Carbonation	7.724 ± 0.470^ab^	7.736 ± 0.526^ab^	8.344 ± 0.495^ab^	7.394 ± 0.459^b^	8.106 ± 0.531^ab^	9.054 ± 0.460^a^
Overall Liking	6.289 ± 0.657^b^	6.510 ± 0.655^b^	9.430 ± 0.455^a^	8.266 ± 0.528^a^	8.995 ± 0.488^a^	9.241 ± 0.522^a^
Face Scale	6.513 ± 0.680^c^	7.276 ± 0.716^bc^	10.247 ± 0.473^a^	8.686 ± 0.594^ab^	8.872 ± 0.482^ab^	9.678 ± 0.507^a^
Perceived Quality	7.399 ± 0.540^bc^	6.923 ± 0.604^c^	9.369 ± 0.443^a^	8.252 ± 0.507^abc^	8.640 ± 0.467^ab^	9.353 ± 0.495^a^

*Note*: The data represents the average of sensory attributes related attributes ± standard error (*n* = 50). The significant letters in each row indicate significant statistical differences between beer samples, according to the method of Least Significant Difference (LSD, *p* < .05). Sample abbreviations are displayed in Figure 1.

Beer samples were obtained from the local market, and unopened bottles of these beers were transported to the participants' home locations for sensory evaluations, and to the Tecnologico de Monterrey Biotechnology Center laboratories for physicochemical testing. As indicated in the following sections, food engineering students administered the sensory tests at‐home, and beer brands were unknown to consumers.

### 2.1 Sensory analysis performed by consumers

Consumer tests were conducted during the pandemic isolation period by students enrolled in the TA2010 Sensory Evaluation courses from Tecnologico de Monterrey. Evaluation kits were picked up by students at a central location. The test kits included commercial beer samples at room temperature (25°C), unsalted crackers, and disposable 1 oz (30 mL) cups. Students who applied the sensory tests had access to frequent beer consumers who lived in Monterrey. Before the sensory analysis, zoom sessions were carried out to instruct students on the test logistics. Sample handling included storing the samples in their home refrigerators (approx. 4°C) and covering bottles with aluminum foil to avoid biases by consumers' knowledge of the beer brands. Beer samples (30 mL) were evaluated at home by 50 consumers aged 20–76 years old (74% male, 26% female). The mean age was 35.7 years old, with a population distribution that included 62% in the age range 20–31.4 years old, 22% in the range of 42.8–54.2 years old, and 16% in the range of 54.3–76 years old.

Sensory responses were recorded in digital questionnaires using RedJade Sensory Software (RedJade Sensory Solutions, LLC). Sensory questionnaires included affective questions to assess overall and attribute liking using 15 cm non structured hedonic scales and check‐all‐that‐apply (CATA) emoji selection questions (Table S1). It also included JAR questions to assess the optimum levels of foam height and bitterness attributes.

**FIGURE 1:**
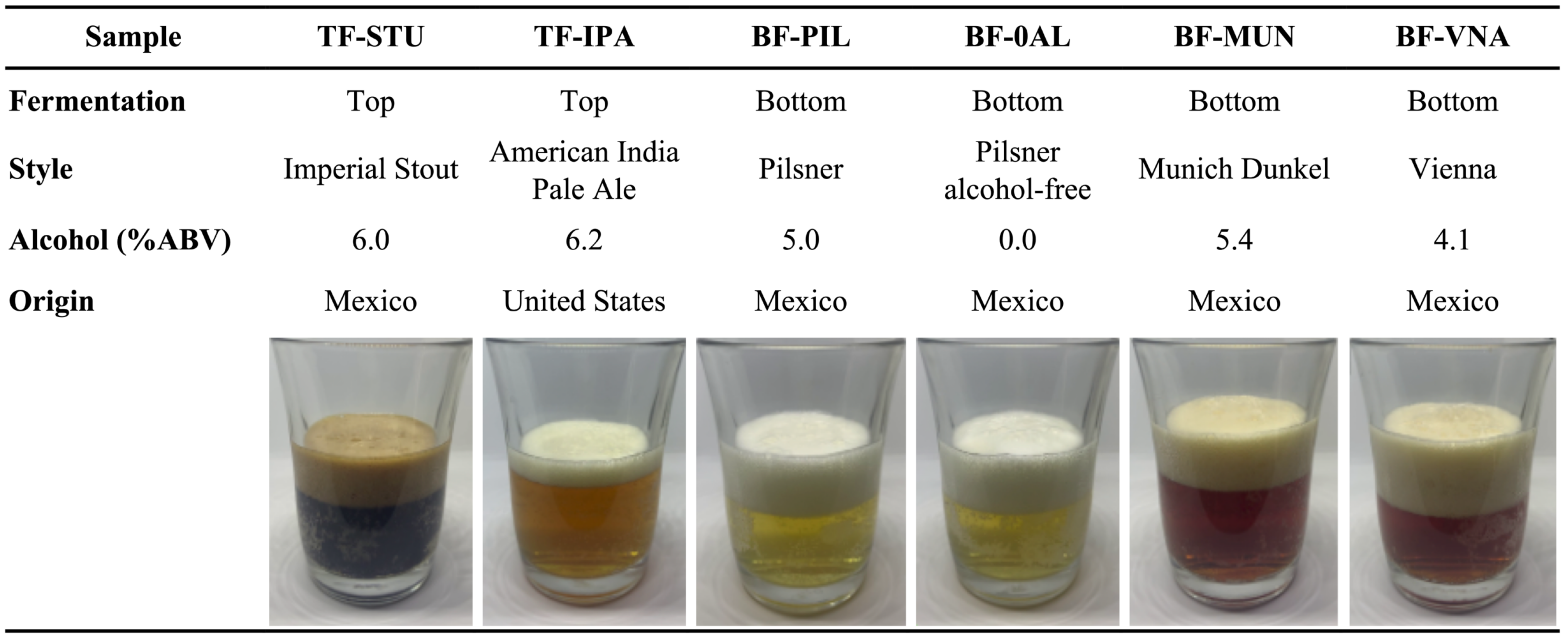
Characteristics of the selected beer samples commercialized in Mexico.

To ensure consistency in the evaluation of visual attributes, a video of beer pouring into a glass (Figure 1) was used. This was followed by tasting the beer samples and answering questions related to aroma, flavor, and emotional attributes (Table S1). The study received approval from the sensory evaluation ethics committee of the Biotechnology Graduate Program, School of Engineering and Sciences at Tecnologico de Monterrey, Campus Monterrey, Mexico (Ethics ID: CSER‐DBT‐0002). Participants recorded their informed consent at the start of each session.

### 2.2 Physicochemical analysis

Before performing the chemical analysis, beer samples were stored at 21°C (20%–25% relative humidity). Each bottle was opened under dark conditions and subjected to a two‐step degassing procedure. First, beer bottles were manually stirred for 10 min until the foam was stabilized. Then, the samples were degassed using an ultrasonic sonicator (5800, Bransonic® CPX) for 90 min at a frequency intensity of 40 kHz. The temperature fluctuated between 22°C and 32°C due to the mild heating effects of the high frequencies. After degassing, aliquots were collected for bitterness analysis (20 mL) and chromatographic analysis (4 mL). The samples were nitrogen‐flushed and stored at −80°C until further evaluation. The degassed samples were also used to determine pH, acidity, color, total soluble solids (TSS), and density. All physicochemical analyses were conducted in triplicates. Instrumental color was measured using a spectrophotometer (Genesys 10SUV, Thermoelectron) at 430 nm, in accordance with the Standard Reference Method (°SRM) color scale (Figure S2) (ASBC, 2018b). Density was assessed based on weight and volume (10 mL). TSS was expressed as °Brix and was measured using a digital refractometer (AT‐PAL‐3, Atago). pH was measured with a potentiometer (Orion Star Series, Thermo Scientific) at room temperature (21°C), and acidity was expressed as milligrams of lactic acid (LA) per liter of beer, as described by ASBC (2018a). Beer bitterness was determined spectrophotometrically (Fisher Chemical) at 275 nm in isooctane extracts and expressed as international bitterness units (IBU) as mentioned in the official beer ASBC methods (ASBC, 2018c).

### 2.3 Characterization of iso‐*α*‐acids (IAA) and hordenine

Individual IAA and hordenine were analyzed as described in Gonzalez Viejo et al. (2020). For IAA determinations, beer samples were prepared by filtering 1.5 mL with a 0.22 μm PTFE syringe filter and directly injecting to an Acquity Ultra‐Performance Liquid Chromatography (UPLC, Waters) coupled to Diode‐Array Detector (DAD), monitoring at 270 nm. The A mobile phase consisted of 5 mM ammonium acetate in 20% ethanol (pH 9.95) and acetonitrile/ethanol (60:40 v/v) as phase B. The solvent flow rate was 0.4 mL·min^−1^, using gradients of 0%–95% B by 65 min, followed by 20 min re‐equilibration. The injection volume was of 10 μL and IAA were separated using a Zorbax Extend C‐18 column (100 × 3 mm, 3.5 μm particle size, Agilent) kept at 35°C. The iso‐*α*‐acids quantification was performed with calibration curves in a range of 50–250 ppm of commercial standards of trans‐iso‐*α*‐acids in dicyclohexylamine (DCHA) obtained from the American Society of Brewing Chemists. The profile of iso‐*α*‐acids in their cis and trans isomers (cis/trans‐iso‐cohumulone, cis/trans‐iso‐humulone, and cis/trans‐iso‐adhumulone) was reported.

For hordenine determinations, samples were diluted twice to reach a dilution factor of 500. Dilution I (Dil I): 50 μL of the degassed beer was mixed with 450 μL of water acidified with 0.1% formic acid (CH_2_O_2_). Dilution II (Dil II): 20 μL of Dil I was mixed with 980 μL of water acidified with 0.1% CH_2_O_2_. The final dilution was filtered with polyvinylidene difluoride (0.2 μm) for injection into the chromatography equipment. Hordenine separation and quantification were conducted in a Quattro Premier XE Micromass UPLC–MS/MS system (Waters) equipped with a triple quadrupole mass spectrometer (QQQ‐MS) connected to an Acquity UPLC (Waters) with electrospray ionization (ESI) source in positive mode. Hordenine was analyzed in the multiple reaction monitoring (MRM) mode of m/z 165.95:121 and Masslynx 4.1 software (Waters) was used for data acquisition and instrument control. The flow rate was kept constant at 0.5 mL·min^−1^ with an injection volume of 10 μL into a high strength silica (HSS T3 C18) column (2.1 mm × 100 mm, 1.8 μm particle size) maintained at 50°C. The mobile phases consisted of 0.1% formic acid in water (solvent A) and 0.1% formic acid in 70% acetonitrile and 30% methanol (solvent B) with 5.2 min total gradient from 5% to 100% of phase B, followed by 1.4 min re‐equilibration at starting conditions (5% B). Nitrogen was used as the desolvation gas (400 L·h^−1^). The selected ion monitoring conditions were set as capillary voltage 2.5 kV, source temperature 120°C, and desolvation temperature 400°C. All determinations were conducted in triplicates. For the quantification, a calibration curve (0.0025–0.025 ppm) was generated using a 1000 ppm stock solution of a commercial standard (Sigma Aldrich) prepared in 0.1% CH_2_O_2_.

### 2.4 Headspace volatile compounds

Headspace volatile compound profiles were obtained following the methodologies described by Attchelouwa et al. (2020) and Gonzalez Viejo et al. (2019) with minor modifications. Beer samples (10 mL) were stored at 4°C overnight in vials. Prior to analyses, samples were conditioned at room temperature (25°C), and sodium chloride (2 g) was added. Viales were securely sealed with PTFE‐silicon septum caps and placed in a heated water bath (50°C) with magnetic agitators. Adsorption of volatile compounds into the polyacrylate fiber (85 μm, fused silica, 24 Ga, white) were conducted with a manual SPME holder (Supelco Inc.) inserted into the headspace of each sample for 15 min.

Following each extraction, the fiber was manually inserted into the gas chromatograph (GC) injector and thermally desorbed at 200°C for 4 min. Injections were carried out with a 10:1 split ratio. Analyses were performed using a Perkin Elmer GC Clarus 690 gas chromatography equipment coupled with a Clarus SQ8T mass spectrometer (PerkinElmer). Separation was achieved using an HP‐5MS capillary column (30 m × 0.25 mm × 0.25 μm) from Agilent Technologies, Inc., with helium as the carrier gas at a flow rate of 1 mL/min. The oven temperature gradient was set to initiate 50°C for 3 min, followed by a ramp of 10°C/min to 200°C with held for 3 min, and a second ramp at 10°C/min to 250°C held for 4 min. The MS analysis operated in the electron ionization (EI) mode (70 eV) and the full scan mode over a mass range of m/z scan = 30–450 amu. Interface and ion source temperatures were maintained at 210°C. Data acquisition was carried out with Turbomass software (Perkin Elmer, version 6.1.0.1963). The volatile flavor profiles were calculated as relative peak area percentages (%RPAs). The % RPAs were calculated as the ratio of the area of each peak to the sum of all the areas of the identified peaks in each beer sample (Attchelouwa et al., 2020; Rodrigues et al., 2008).

Peaks were assigned putative identities by comparing mass spectrums to those of NIST libraries (match >80%) (National Institute of Standards and Technology, Gaithersburg, MD, United States). Sensory aroma descriptors for the putative compounds were obtained from previously published literature and the NIST database (Gonzalez Viejo et al., 2019; Liu & Quek, 2016; Praia et al., 2022; The Good Scents Company, 2022).

### 2.5 Statistical analysis

Utilizing XLSTAT 2022.2.1 (Addinsoft) and analysis of variance (ANOVA) for parametric data with mean separation and Least Significant Difference (LSD) post hoc test (*α* = .05), data from consumer sensory assessment was examined. Data from the Just‐About‐Right (JAR) scale were subjected to penalty analyses using Microsoft 365 Excel 16.61 (Microsoft), as reported by Walker (2016). ANOVA was used to evaluate the physicochemical data, and LSD was used to separate the means (*α* = 0.05) using Minitab 19 (Minitab Inc). Physicochemical and sensory variables, including check‐all‐that‐apply (CATA) emojis, were significantly correlated (*p* < .05) using a MathLab® R2021a code (Mathworks, Inc). Physicochemical characteristics, volatile chemicals, sensory consumer acceptability, and CATA responses were all included in the multiple factor analysis (MFA) carried out using XLSTAT 2022.2.1 (Addinsoft) (Addinsoft, 2022).

## 3 RESULTS

### 3.1 Consumer acceptability

Results from the sensory acceptability evaluation showed significant differences (*p* < .05) among most attributes, except for foam stability, aroma, and carbonation (Table 1). Regarding visual sensory attributes, Vienna‐style (BF‐VNA) beer received significantly lower acceptability scores for foam height, whereas TF‐STU, a dark‐colored beer, had significantly lower color liking than the other samples. Overall liking was rated higher for bottom‐fermented beers compared to top‐fermented ones, with BF‐PIL receiving the highest hedonic score of 9.4. Bitterness acceptability data indicated that bottom‐fermented beers were better liked, with hedonic values ranging from 8.1 to 9.1 (Table 1). Acidity and sweetness were also significantly higher for the Pilsner‐style beers (BF‐PIL). Responses from the face scale and perceived quality questions also indicated that the most liked samples were BF‐PIL and BF‐VNA, which align with their hedonic scale values. On the other hand, the two top‐fermented beers (TF‐STU and TF‐IPA) received significantly lower overall liking scores, with TF‐STU having the lowest average liking score of 6.3. This trend was consistent with the scores of specific attributes, including flavor, sweetness, acidity, bitterness, and the face scale (Table 1).

### 3.2 Consumer responses to just‐about‐right (JAR) scale and penalty analysis

**FIGURE 2:**
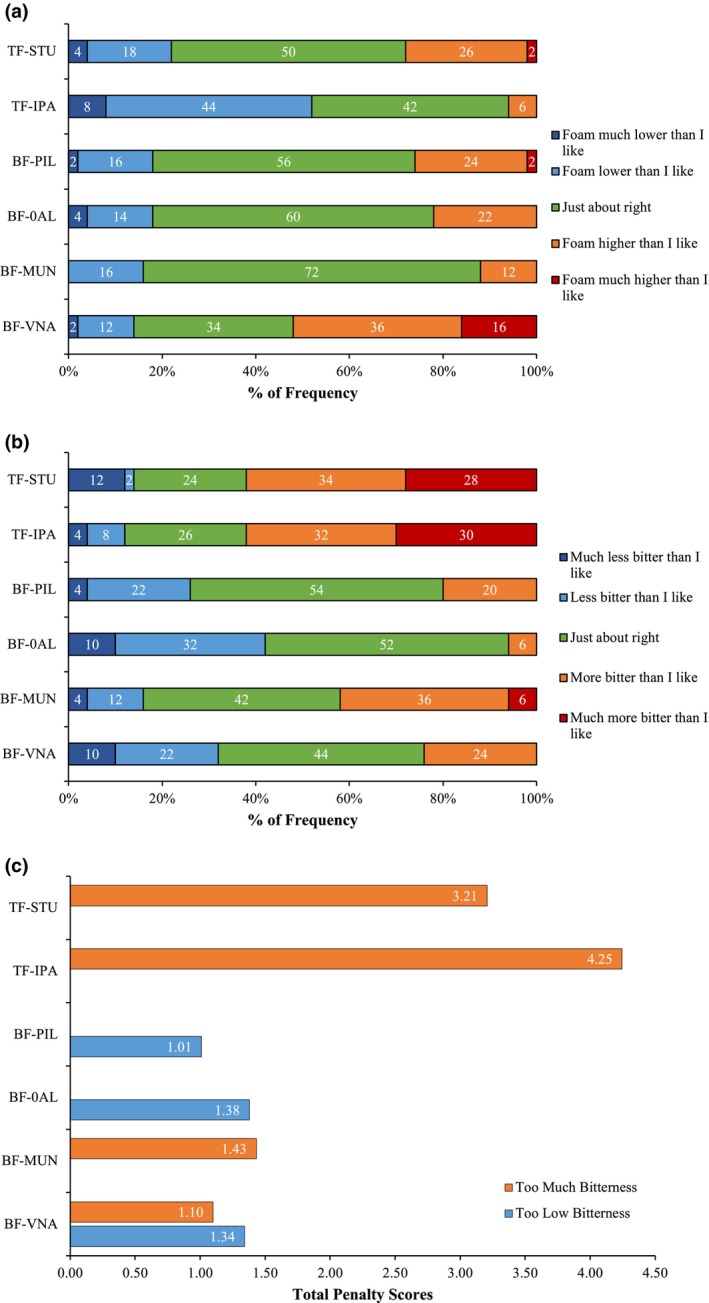
Just‐about‐right (JAR) data for foam height (a) and bitterness (b) are presented as percentages. The penalty analysis for bitterness (c) is based on the JAR test and overall liking score.

Among the beer samples, BF‐MUN (Figure 2a) had the highest JAR frequency for the optimum foam height, featuring a medium‐height foam when compared visually to the other samples. After watching the pouring video, 52% of consumers responded that the foam of the sample TF‐IPA was too low. Conversely, for BF‐VNA, 52% of responses indicated that the foam was too high.

**FIGURE 3:**
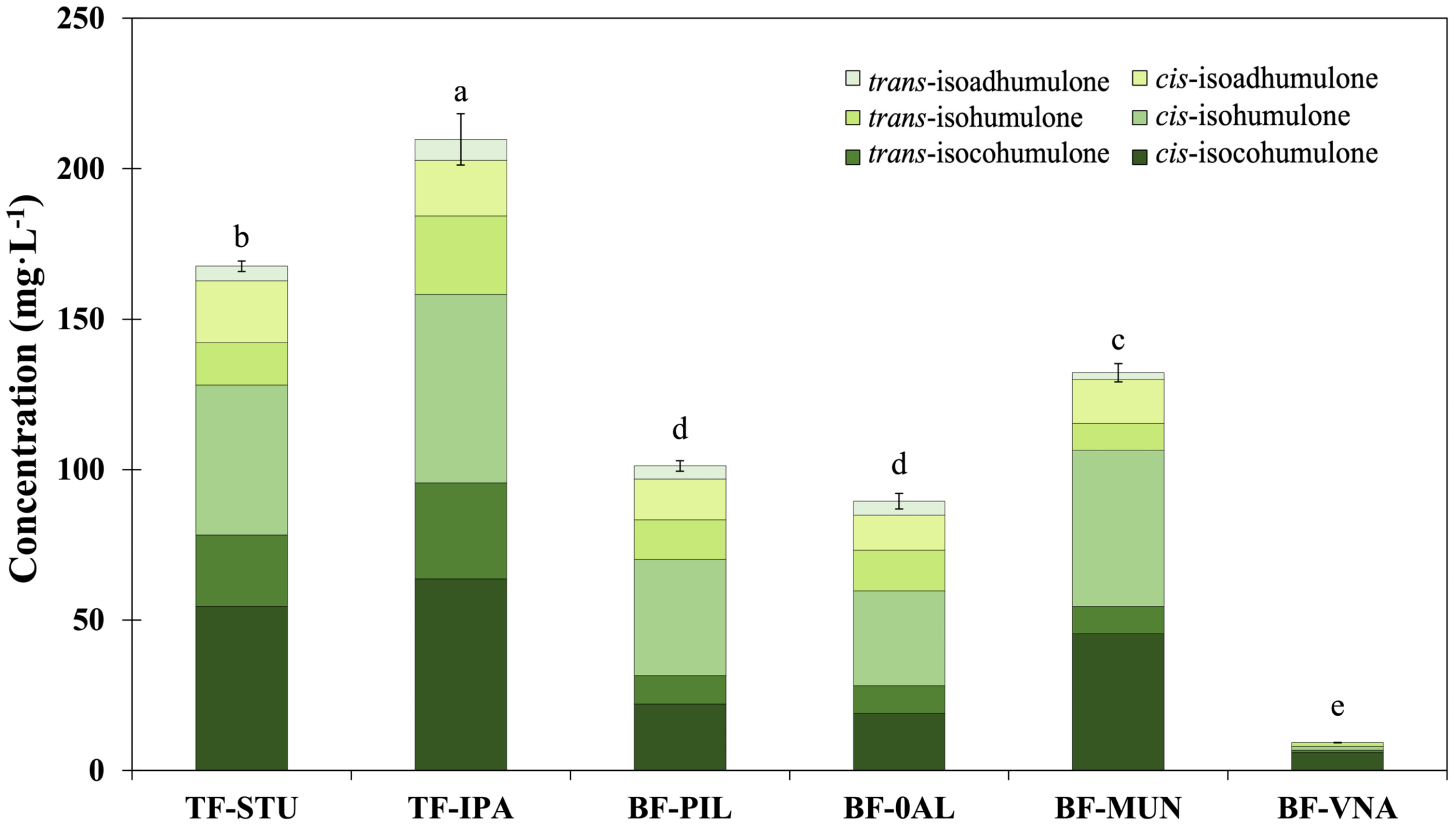
Concentrations of iso‐*α*‐acids (IAA) in six commercial beer samples, including two top‐fermented and four bottom‐fermented samples. Sample details and abbreviations are defined in Figure 1. The data corresponds to the mean values of individual IAA (*n* = 3) and the error bar to total IAA. Different letters indicate significant differences between total IAA beer samples according to the Least Significant Difference method (LSD, *p* < .05).

The JAR scale was also utilized to assess the desirable bitterness levels (Figure 2b). The Lager Pilsner‐style beer, BF‐PIL, received the highest JAR bitterness responses, with 54% of consumers considering its bitterness level as optimum. In contrast, for samples TF‐STU and TF‐IPA, 62% of consumers evaluated their bitterness as too high, leading to an overall liking penalization of 3.21 and 4.25 points on the hedonic scale, respectively (Figure 2c). Interestingly, the zero‐alcohol beer (BF‐0AL) was evaluated as too low in bitterness by 42% of consumers, even though it had similar IBUs and IAA as its alcoholic counterpart, BF‐PIL (Table 2, Figure 3). The perceived lower bitterness of the nonalcoholic beer resulted in a significant hedonic penalty of 1.38 for being less bitter than the desirable level.

**TABLE 2:** Physicochemical properties of six beer brands commercialized in Mexico.

Sample	Color (°SRM)	TSS (°brix)	Density (g·mL^−1^)	Acidity (LA) (mg·L^−1^)	pH	Bitterness (IBU)	Hordenine (mg·L^−1^)
TF‐STU	150.877 ± 1.429^a^	8.600 ± 0.000^a^	1.011 ± 0.004^b^	2.712 ± 0.012^a^	4.392 ± 0.003^c^	37.417 ± 0.337^b^	10.75 ± 1.352^a^
TF‐IPA	010.283 ± 0.012^d^	7.500 ± 0.058^b^	1.015 ± 0.001^ab^	2.724 ± 0.033^a^	4.496 ± 0.001^a^	53.800 ± 0.231^a^	07.25 ± 0.511^b^
BF‐PIL	003.560 ± 0.000^e^	5.467 ± 0.033^d^	1.020 ± 0.002^a^	1.776 ± 0.049^c^	4.454 ± 0.003^b^	16.817 ± 0.142^d^	04.92 ± 0.432^c^
BF‐0AL	003.470 ± 0.000^e^	5.400 ± 0.000^d^	1.021 ± 0.001^a^	1.674 ± 0.063^c^	4.357 ± 0.014^d^	15.500 ± 0.076^e^	05.26 ± 0.443^bc^
BF‐MUN	020.117 ± 0.065^b^	6.567 ± 0.033^c^	1.018 ± 0.001^a^	2.106 ± 0.018^b^	4.264 ± 0.002^e^	19.250 ± 0.275^c^	02.52 ± 0.445^d^
BF‐VNA	016.480 ± 0.291^c^	5.000 ± 0.000^e^	1.016 ± 0.001^ab^	1.110 ± 0.016^d^	4.182 ± 0.001^f^	10.833 ± 0.073^f^	01.61 ± 0.445^d^

*Note*: The data correspond to the average of physicochemical variables ± standard error (*n* = 3). The significant letters in each column indicate significant statistical difference between beer samples, according to the method of Least Significant Difference (LSD, *p* < .05). Sample abbreviations were displayed in Figure 1.

Abbreviations: °SRM, Standard Reference Method; IBU, International Bitterness Units; LA, lactic acid; TSS, Total Soluble Solids.

### 3.3 Chemometric analyses

#### 3.3.1 Physicochemical results

All physicochemical parameters exhibited significant differences (*p* ≤ .05) evaluated parameters except for density (Table 2). Spectrophotometrically, TF‐STU was the darkest beer (Figure 1), consistent with its higher °SRM color value of 150.88, followed in darkness by the BF‐MUN and BF‐VNA samples with had °SRM values of 20.12 and 16.48, respectively; these values correspond to an amber‐reddish tone (Table 2). The TF‐IPA sample had a °SRM color value of 10.28, which translates into a bright golden color. The other two lager‐type samples, BF‐PIL and BF‐0AL, presented values around 3.5 °SRM, interpreted as a straw‐yellow color. Both top‐fermented beers had higher values of total soluble solids values (7.5–8.6 °Brix) than every bottom‐fermented beers (5.0–6.6 °Brix). A similar trend was also observed for acidity and bitterness measurements, as the top‐fermented samples were more acidic (2.71–2.72 mg·L^−1^) and exhibited higher bitterness levels (37.40–53.80 IBU) than bottom‐fermented samples with acidic values ranging from 1.67–2.1 mg·L^−1^ and bitterness from 10.80–19.25 IBU.

#### 3.3.2 Iso‐*α*‐acids (IAA) profile

The characterization of IAA included both *cis* and *trans* isomers in the profiles of individual compounds (Figure 3). For both types of fermentation, the *cis* isomers levels were higher than the *trans* isomers, accounting for 69%–85% of the total IAA pool.

The two top‐fermented beers (Ales) had significantly higher concentrations of total IAA levels (167.58 and 209.72, for TF‐STU and TF‐IPA, respectively) than the bottom fermentation samples (Figure 3). Within the lager beers, BF‐MUN contained the highest total IAA levels (132.2 mg·L^−1^), followed the alcoholic and nonalcoholic Pilsners (BF‐PIL and BF‐0AL) with nonsignificantly different values of 101.22 and 89.51 mg·L^−1^. The lowest IAA levels were observed in the Vienna‐style beer (BF‐VNA) at 9.31 mg·L^−1^.

#### 3.3.3 Hordenine content

The top‐fermented beers (Stout and IPA) had significantly higher hordenine concentrations (10.75 mg·L^−1^ and 7.25 mg·L^−1^, respectively) (Table 2). In contrast, BF‐MUN and BF‐VNA contained the lowest hordenine concentrations (2.52 mg L^−1^ and 1.61 mg L^−1^, respectively). The levels of the TF‐IPA and the nonalcoholic Pilsner (BF‐0AL, 5.26 mg·L^−1^) were not statistically different; since the latter contained slightly higher levels than its nonalcoholic (BF‐0AL) counterpart (BF‐PIL), although the values were not significantly different among Pilsners.

#### 3.3.4 Volatile compounds profile results

In the beer samples, 38 distinct volatile chemicals were found and tentatively identified. Table S2 reports the percent contributions of each molecule to the total. Only eleven compounds, including three fatty acids, phenylethyl alcohol, isoamyl alcohol, and six esters, were discovered to be present in all samples. Octanoic acid, isoamyl alcohol, and ethyl decanoate made up the three primary volatiles in stout beer (TF‐STU), which together accounted for 58.4% of the molecules in the profile. Isoamyl decanoate, ethyl myristate, ethyl palmitate, butylated hydroxytoluene, caryophyllene, humulene, and humolol were among the seven compounds discovered only in the headspace of that beer that were present in TF‐STU. It is interesting to note that the main components of the sample TF‐IPA profile were isoamyl alcohol, octanoic acid, phenylethyl alcohol, and linalool, which together made up 59% of the profile. It also contained five unique volatile compounds such as citronellol, 2‐undecanol, methyl geranate, 2‐undecanone and geraniol. Phenylethyl alcohol, phenylethyl acetate, and isoamyl alcohol were the three main volatile substances in the sample BF‐PIL profile, making up 51.87% of the total. Phenylethyl alcohol and octanoic acid made up 48% of the molecules found in the nonalcoholic beer (BF‐0AL), and they were followed by isoamyl alcohol and two phenolics, which were distinct compounds (Table S2). The Munich Dunkel beer style (BF‐MUN) included compounds that were not present in the other samples: 2‐Ethylhexanol, farnesyl acetate, and 2,3‐dihydrofarnesyl acetate. Except for the nonalcoholic Pilsner, all of the beers in the set had a significant amount of isoamyl alcohol in their profiles. Isoamyl alcohol made up about 30% of the headspace volatiles in sample BF‐MUN. Similar to this, it was the primary volatile present (24%) in Vienna beer style (BF‐VNA), which also included four different compounds: citronellyl acetate, 2‐phenylethyl hexanoate, α‐eudesmol, and 1‐decanol.

## 4 DISCUSSION

Since the present work focused on commercial samples, specific details of formulations and processes were not publicly available; however, Figure S2 was constructed using the style characteristics from beer guidelines published by Gatza et al. (2023) and Strong and England (2021). As described in the guidelines, darker styles use specialty malts (i.e., caramel malt and toast malt) or other hop varieties (i.e., Cascade for floral aromas, Citra for citric, and Columbus for herbal aroma) as their main ingredients. Beer styles BF‐VNA, BF‐MUN TF‐STU, and TF‐IPA possibly used those ingredients to enhance their aromatic herbal/floral and caramel notes, as well as their denser bodies (Figure S1). Carbonation level is also described in the style guidelines. Darker beers tend for the lower‐mid carbonation profile, while Pilsner beers tend for a higher carbonation sensory trait. Finally, the use of enzymes for the reduction of dextrins can reduce body, as well as the inactivation β‐amylase may reduce the presence of fermentable sugars (BF‐0AL and BF‐PIL) (Salanță et al., 2020). Both top fermentation beers (TF‐IPA and TF‐STU) shared similar characteristics associated with their styles like turbidity, high alcohol by volume (ABV), herbal and floral aromatic traits, and a high bitterness (Figure 1, Table 2). Bottom fermentation beers (BF‐MUN, BF‐VNA, BF‐PIL, and BF‐0AL) shared characteristics that included low body, grain aroma, low to mid bitterness, and a lower ABV.

### 4.1 Color and foam appearances

**FIGURE 4:**
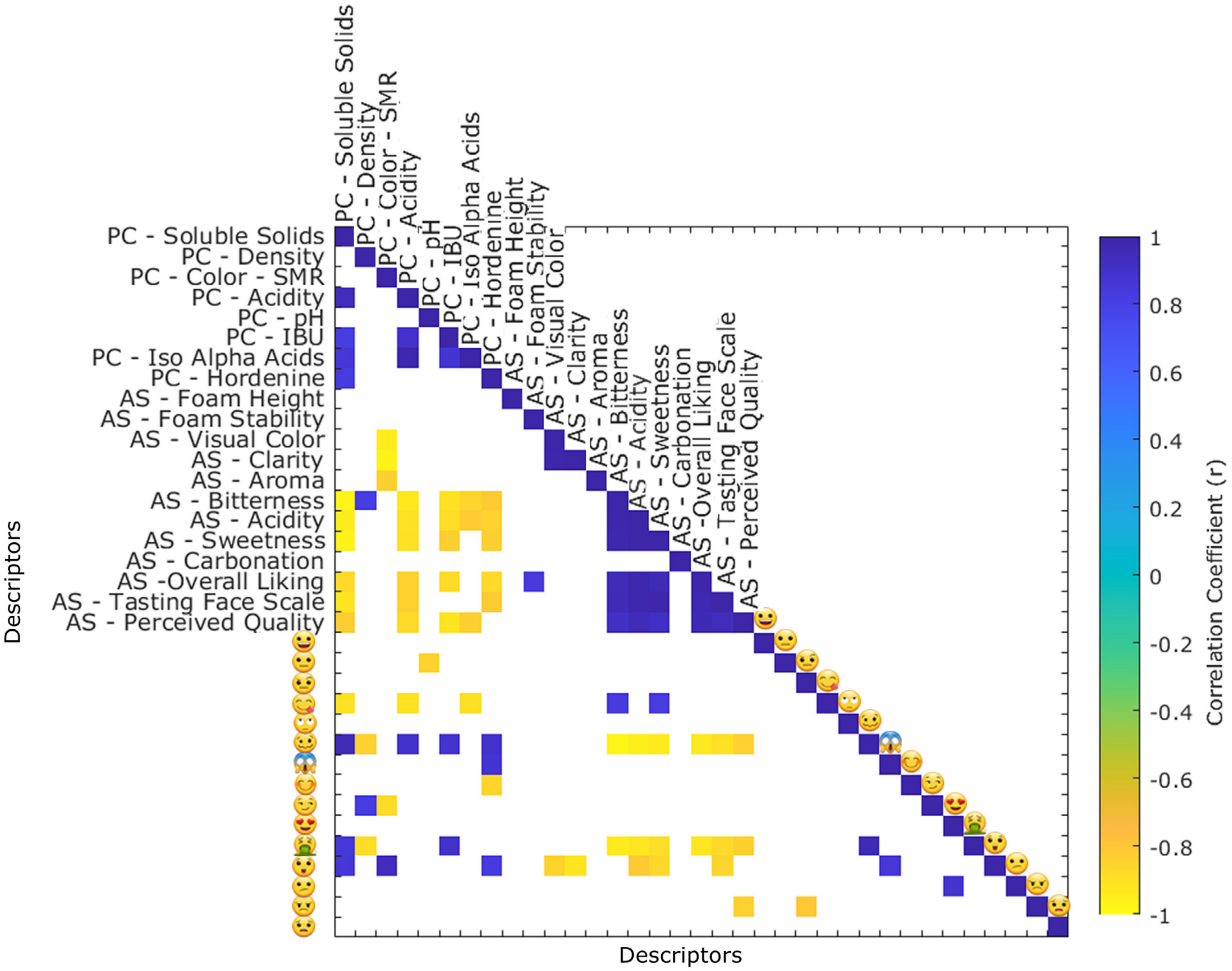
Correlation matrix between the physicochemical parameters (PC), sensory affective responses (AS), and check‐all‐that‐apply (CATA) emojis collected as response variables in six commercial beer samples, including two top‐fermented and four bottom‐fermented samples. The color bar represents significant positive and negative correlations on the indigo and yellow sides, respectively (*p* < .05).

**FIGURE 5:**
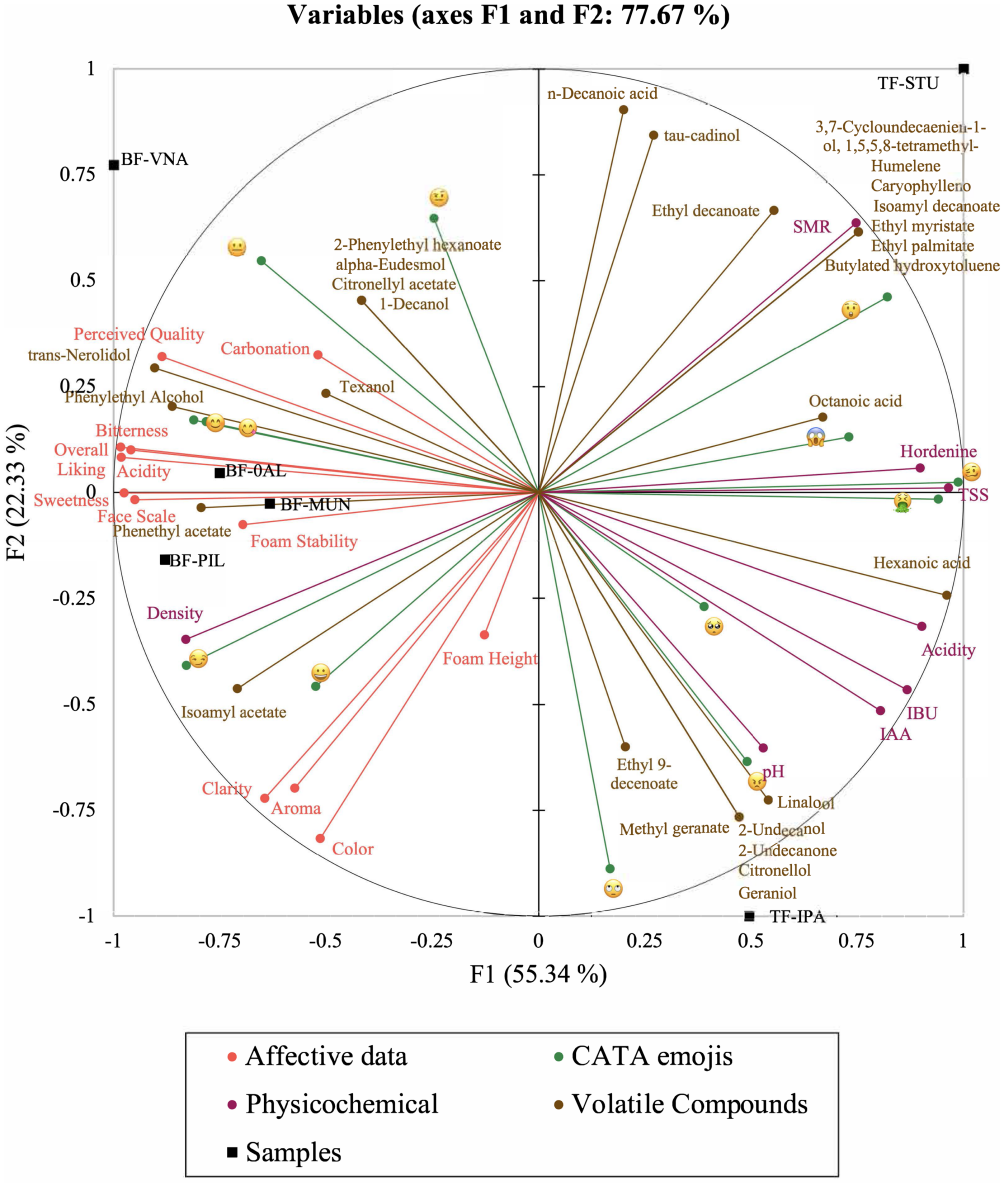
Multiple factor analysis biplots showing physicochemical parameters (in purple), volatile compounds (in brown), sensory responses from consumers (in red), and check‐all‐that‐apply (in green) emojis from consumers for each sample (in black). F1 and F2 represent Factors one and two, respectively.

Color and foam are two visual sensory elements that have been shown to influence consumers' expectations prior to tasting (Van Doorn et al., 2019). In this study, BF‐PIL and BF‐MUN showed greater foam height affective responses (Table 1). The JAR test showed that the largest proportion of answers fell into the ideal category for beers with medium foam height for those same samples (Figure 2a). These results were consistent with those of prior research, which found that Mexican beer drinkers preferred Pilsner bottom fermentation beers (Gonzalez Viejo et al., 2022). While some authors argue that consumer liking is a multifactor reaction, color is another parameter known to impact consumers' expectations throughout the tasting experience (Guinard et al., 1998; Van Doorn et al., 2019). Higher °SRM color intensity beers in this investigation had lower color liking (*r* = −0.95) (Figure 4). Similar findings have been linked to consumer biases with dark‐colored beers, presumably as a consequence of an expectation of reduced thirst satisfaction, or an association with greater bitterness (Betancur et al., 2020; Van Doorn et al., 2019). Since Mexican consumers tend to choose light‐colored beers like Pilsners, which are the most popular beers in Mexico and tend to be less bitter, it is probable that visual color had an impact on the participants in this study, having a predominant preference over light‐colored beers (Gómez‐Corona et al., 2016). However, as evidenced by the high standard error for the visual color liking attribute (Table 1), not all consumers disliked darker beers. It is known that a high degree of response dispersion has been attributed to differences in opinion between novice and expert beer tasters, which is a factor that can affect liking for dark‐colored beers (van Doorn et al., 2019). The physicochemical attributes of color and clarity in the MFA plot were not clearly linked to a specific beer sample in the biplot (Figure 5), but they loaded in the opposite quadrant from the stout beer style (TF‐STU), which was linked by consumers to an emoji of an astonished face (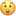), as opposed to the grinning face (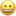).

### 4.2 Bitter and acid chemical molecules

Bitter molecules are well known to significantly influence beer preferences, as well as other attributes like sweetness and carbonation (Betancur et al., 2020). Among the bitter compounds, IAA extracted from hops play a prominent role and are responsible for imparting bitterness, with higher intensity being associated with *cis*‐isohumulone, the main component in five of the beers tested in this study (Figure 3). It is worth noting that other compounds in hops, such as polyphenols and oxidation products, can also impact the overall bitterness profile (Almaguer et al., 2014). The measurement of IBUs involves molecules that absorb at 275 nm wavelength, which means it does not solely represent IAA but also includes oxidized polar compounds from hops and polar compounds from malt (Kishimoto et al., 2022). While IBU values may not provide as specific chromatographic profiles, in this study, they showed the highest inverse correlations with bitterness liking (*r* = −0.91) and overall liking (*r* = −0.88) (Figure 4). Elevated levels of IAA and hordenine also demonstrated a tendency to decrease bitterness hedonic scores (*r* = −0.85 and −0.82 for IAA and hordenine, respectively). Similarly, IBU and IAA levels were inversely correlated with the emojis of perceived quality (*r* = −0.91), woozy face 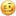 (*r* = 0.88), and face vomiting 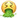 (*r* = 0.90). Likewise, in the JAR scale questions, consumers penalized both top‐fermented beers (Figure 2c), which contained higher IBU, IAA, and hordenine contents (Table 2, Figure 3), and associated them with negative emojis from CATA (Figure 4). Previous studies have also identified low bitterness as a key factor for increasing preference of Brazilian beer consumers (da Costa Jardim et al., 2018). Similar results were reported with Mexican consumers by Gonzalez Viejo et al. (2020), where bitterness disliking were correlated with hordenine level, IBU, and negative emotions (both conscious and unconscious). The same authors suggested that hordenine itself may contribute to the bitterness of beer.

Although consumers were unable to accurately differentiate basic taste sensory attributes, the responses of consumers to the liking of the acidity (Table 1) were significantly and inversely correlated to the physicochemical acidity values (*r* = −0.91) (Table 2). The relationship was also evident in the MFA biplot (Figure 5), wherein less acidic beers, including the bottom‐fermented. Pilsners and Munich Dunkel beer, were shown to have higher acceptability for acidity in the opposite quadrant from the acidity levels.

### 4.3 Higher concentrations of hoppy and fruity headspace volatiles effect on liking

In this study, hop‐derived volatiles were recognized such as linalool, citronellol, geraniol, humulene, caryophyllene, and tau‐cadinol were only discovered in top‐fermented beers (Table S2). Linalool content has been linked to hop taste strength, and it is affected by hop type, amounts utilized, and processing conditions (Sakuma et al., 1991; Steyer et al., 2017). Regarding the current results, Gonzalez Viejo et al. (2019) examined many top‐fermented beers and discovered that linalool was exclusively found in the top‐fermented samples. Linalool and other hop‐derived compounds have been linked to the classic hoppy aroma in beer (Almaguer et al., 2014).

Interestingly, both top‐fermented beers were found on the right side of the MFA biplot in Figure 5; the Stout beer (TF‐STU) was in the right‐top quadrant, possibly due to its more complex and hoppy volatile profile, and the TF‐IPA was in the bottom quadrant, most likely due to its high IBU and IAA content. The TF‐STU was detected in the same sample as the astonished face (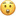), ethyl decanoate, tau‐cadinol, n‐decanoic acid, and other volatile compounds.

Both top‐fermented beers had considerably higher values in the majority of physicochemical parameters, including SRM Color, TSS, IBUs, IAA, and hordenine, which affected their position in the right side of the MFA plot (Figure 5). Since the samples' overall consumer acceptability was lower, they were located in the opposite quadrant from liking. The positive side of factor 1 (F1) was represented by emojis of negative or unfamiliar sensory perceptions, such as a woozy face 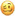 (*r* = 0.99), and the negative side of factor 2 (F2) was primarily represented by a face with rolling eyes 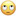 (*r* = −0.89) in the regions where top‐fermented beers were placed.

Prior studies did not show an obvious a differentiation by fermentation type as this research, probably because the physicochemical parameters assessed only involved soluble solids, IAA, acidity, phenolic compounds, and antioxidant activity (Baiano & Terracone, 2013). Additional physicochemical factors such as IBUs, volatiles, and hordenine may have helped to explain the discrepancies between the samples of this study. Hordenine content has been observed to be greater in top‐fermented beers, which is consistent with the current investigation (Brauers et al., 2013; Gonzalez Viejo et al., 2020).

In the present study, the BF‐VNA was the only beer with citronellyl acetate in its profile, which is known to have a scent of flowers, greens, roses, and citrus (The Good Scents Company, 2022). Notably, this beer had an overall liking score of 9.2, and loaded in the quadrant of preferred samples (Figure 5). Some of its characteristic volatile compounds, including citronellol and geraniol, undergo acetylation during fermentation (King & Dickinson, 2006; Steyer et al., 2017), therefore, were likely key contributors to the BF‐VNA sample distinct aroma profile. The volatile profile of beers can be shaped by several factors including the wort's ingredients, fermentation conditions, yeast strain, and environmental variables like temperature, pressure, and oxygen content (He et al., 2014). Volatile compounds emerge as by‐products of the metabolic transformation of wort components, essential for yeast growth and development (Olaniran et al., 2017). Specifically in bottom‐fermented beers, the yeast metabolism plays a significant role in the volatile profile. Bottom fermenting strains follow metabolic pathways that produce fewer flavor compounds when compared to top‐fermenting yeasts, resulting in a less complex volatile profile (Dack et al., 2017; Paszkot et al., 2023). Furthermore, the ingredient selection, particularly the use of subtler malts and hops, contrasts with the aromatic and diverse ingredients in top‐fermented beers, further contributing to the distinctive volatile profiles of bottom‐fermented beers (Olaniran et al., 2017; Paszkot et al., 2023).

**FIGURE 6:**
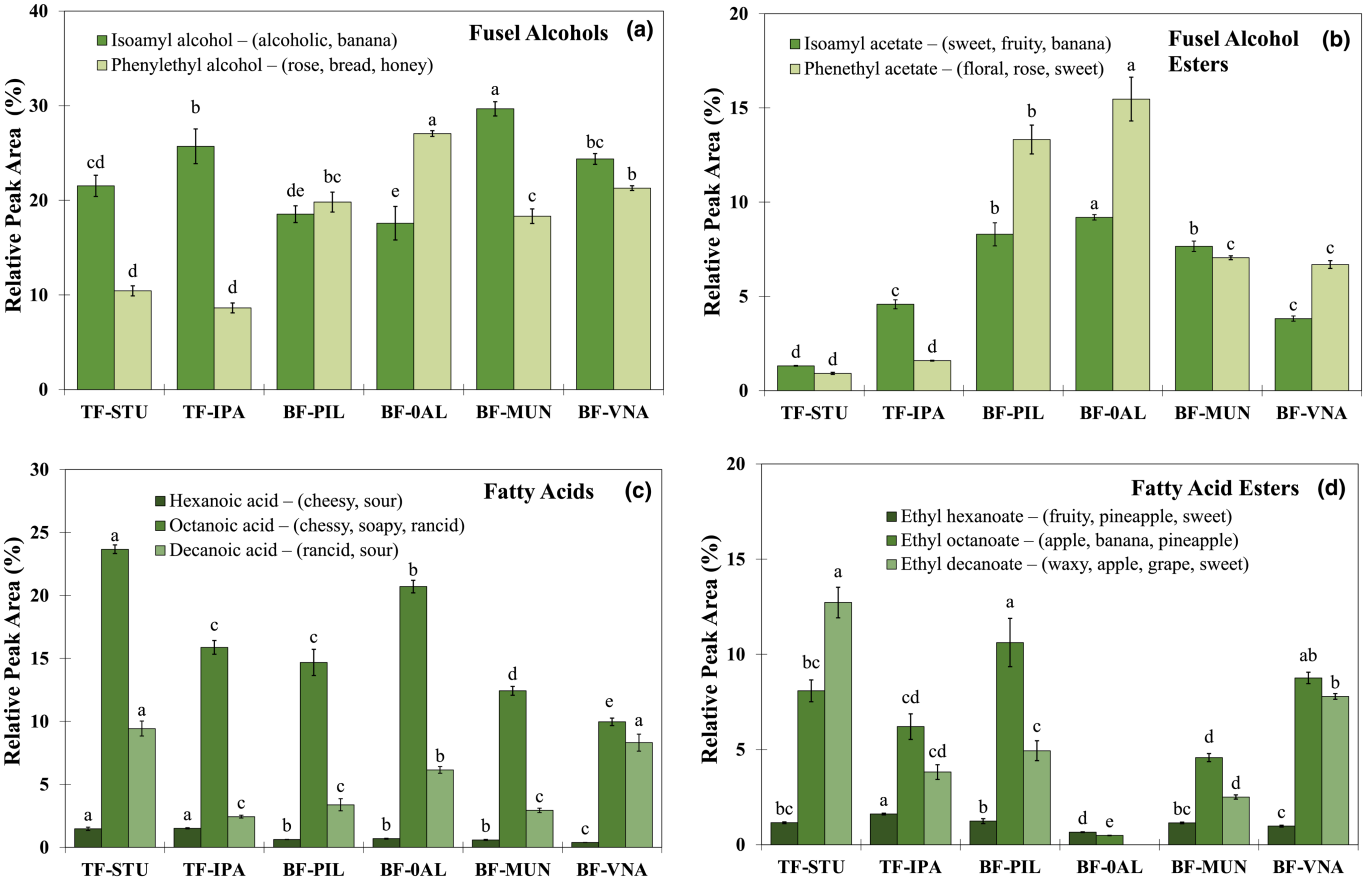
Volatile compound profiles (%) in six commercial beer samples, including two top‐fermented and four bottom‐fermented samples. Sample details and abbreviations are defined in Figure 1. The data represent the mean value for each individual compound present in each sample (*n* = 3). The letters above the graph bars indicate significant statistical differences between beer samples for the same volatile compound, which were determined using the method of Least Significant Difference (LSD, *p* < .05). Volatile compounds were identified by comparing the mass spectrum of the compound to the mass spectrum libraries of the National Institute of Standards and Technology (NIST; National Institute of Standards and Technology, Gaithersburg, MD, United States), with a match of >80% considered for identification.

A lower °Brix value, lower acidity, and reduced bitterness further distinguished the Vienna samples from Pilsners (Table 2). Figure 6a,b show that BF‐VNA had lower levels of fruity volatiles such as isoamyl acetate and significantly greater levels of isoamyl alcohol than Pilsners (24.5% vs. 17.6%–18.6%, respectively). Additionally, the Pilsners and Munich Dunkel shared greater amounts of isoamyl acetate (7.6%–9.2%, respectively), which is known to impart fruity, banana‐like, estery aromatic characteristics (Figure 6b), with the most popular beers. Additionally, phenethyl acetate, which is known to impart floral and sweet flavors, was present in them at much greater levels (13.4%–15.5%) than in the other beers (Figure 6b).

However, larger concentrations of fruity notes also led to variances in their emotional reactions. The current investigation indicated that Northern Mexico customers favor bottom‐fermented beers low in bitterness. Belgian beers of spontaneous fermentation with cherry and raspberry notes were tested in a prior study with Mexican consumers, and the results showed that these beers produced significantly higher responses in the overall liking hedonics (10.04–10.35) than the Lager Pilsner‐style beer they tested, which had a liking value of 8.30 (Gonzalez Viejo et al., 2019). The findings suggested that the beers in each sample set may affect how much a customer likes a certain beer type. The overall liking ratings for the Pilsner beers (BF‐PIL) and those without alcohol (BF‐0AL) in this study were 9.43 and 8.27, respectively. The identical Pilsner beers included in the current investigation were examined with and without alcohol in a previous study that employed biometrics to analyze consumer reactions, and hedonic values of 10.09 and 8.75, respectively, were achieved (Gonzalez Viejo et al., 2023). They conducted their survey in a central area after the pandemic, while the current research was conducted at home during the pandemic, and responses were strikingly comparable, confirming Mexican consumers' strong preference for the Pilsner beer type. Fatty acid concentrations in the headspace are also known to influence flavor profiles. Their aroma descriptors, as detailed in literature in Table S2, are often more associated with fatty notes rather than fruity notes. In the Pilsner (BF‐0AL) and Stout (TF‐STU), both of which are nonalcoholic beverages, octanoic acid concentrations were noticeably greater (Figure 6c). According to Steyer et al. (2017), it is known that volatile fatty acids, which are responsible for fatty, sour, cheesy, and rancid smells, are created by yeast lipid metabolism. They may be esterified in the presence of ethanol to create substances with more appealing sensory descriptors, such as ethyl octanoate, which imparts aromatic notes of apple, banana, and pineapple. The highly regarded Pilsner and Vienna beers had much larger amounts of this desired volatile (ethyl octanoate), but the nonalcoholic Pilsner had relatively lower quantities (Figure 6d). The nonalcoholic beer's volatile profile may have been impacted by the manufacturing process; nonetheless, its acceptability values were higher than those of top‐fermented beers, even though they were not significantly different from those of its alcoholic counterpart (8.27 vs. 9.43, respectively) (Table 1).

## 5 CONCLUSIONS

This study found that variations in the chemical composition of beer samples significantly influenced their acceptability among Mexican consumers. Beers with the sensory perception of high bitterness associated with the top‐fermented beers, Stout, and American IPA styles, had lower liking scores and negative emotions, attributed to elevated levels of IBUs, IAA, and hordenine. Ales also had lower concentrations of fusel alcohol esters, related to fruity aromatic notes, and higher amounts of bitter and acid molecules. Conversely, bottom‐fermented beers (Lagers), particularly Pilsners and Munich Dunkel, were preferred, featuring low bitterness and high levels of compound aromatics and fruity esters. Low acidity in samples correlated with higher liking scores for the sensory acidity attribute. Nonalcoholic and alcoholic Pilsner beers had similar overall liking scores, but their hedonic values were lower, linked to their volatile profile with minimal fatty acid esters. In conclusion, this study underscores the importance of considering the chemical composition of beers, especially bitter molecules, in understanding consumer preferences, while also highlighting the significance of hordenine, acidity, and volatiles for a comprehensive insight into liking.

## AUTHOR CONTRIBUTIONS

**Perla A. Ramos‐Parra:** Data curation (equal); formal analysis (equal); methodology (equal); visualization (equal); writing – original draft (equal). **Irma C. De Anda‐Lobo:** Data curation (equal); formal analysis (equal); methodology (equal); visualization (equal); writing – original draft (equal). **Claudia Gonzalez Viejo:** Conceptualization (equal); investigation (equal); methodology (equal); project administration (equal); resources (equal); visualization (equal); writing – review and editing (equal). **Raúl Villarreal‐Lara:** Conceptualization (equal); methodology (equal); project administration (equal); resources (equal); visualization (equal); writing – review and editing (equal). **Jorge Abraham Clorio‐Carillo:** Data curation (equal); formal analysis (equal); methodology (equal); writing – original draft (equal). **Luis Martín Marín‐Obispo:** Data curation (supporting); formal analysis (supporting). **Diana Jessica Obispo‐Fortunato:** Data curation (supporting); formal analysis (supporting). **Zamantha Escobedo‐Avellaneda:** Methodology (equal); visualization (equal); writing – review and editing (equal). **Sigfredo Fuentes:** Conceptualization (equal); investigation (equal); methodology (equal); resources (equal); visualization (equal); writing – review and editing (equal). **Esther Pérez‐Carillo:** Methodology (equal); visualization (equal); writing – review and editing (equal). **Carmen Hernandez‐Brenes:** Conceptualization (equal); investigation (equal); methodology (equal); project administration (equal); resources (equal); visualization (equal); writing – review and editing (equal).

## CONFLICT OF INTEREST STATEMENT

The authors declare no conflicts of interest.

## Supporting information


Data S1.

## Data Availability

The data that support the findings of this study are available from the corresponding authors upon reasonable request.
