# Supplementary material for: Consumer insights into the at‐home liking of commercial beers: Integrating nonvolatile and volatile flavor chemometrics
Source: Food Sci Nutr. 2024 Mar 7;12(6):4063–75. doi: 10.1002/fsn3.4066 (PMC11167190; doi:10.1002/fsn3.4066)
Supplement: Supplementary file 1 — Data S1. [file FSN3-12-4063-s001.pdf]

Supplementary Table 1. Questions presented in the sensory consumer evaluation using RedJade

Attribute	Responses	Type of Scale
Foam height	Dislike extremely – Like extremely	15-cm non structured
Foam height	Much less than I like – Much more than I like	5-point just about right
Foam stability	Dislike extremely – Like extremely	15-cm non structured
Color	Dislike extremely – Like extremely	15-cm non structured
Clarity	Dislike extremely – Like extremely	15-cm non structured
Aroma	Dislike extremely – Like extremely	15-cm non structured
Bitterness	Dislike extremely – Like extremely	15-cm non structured
Bitterness	Much less than I like – Much more than I like	5-point just about right
Acidity	Dislike extremely – Like extremely	15-cm non structured
Sweetness	Dislike extremely – Like extremely	15-cm non structured
Carbonation	Dislike extremely – Like extremely	15-cm non structured
Overall liking	Dislike extremely – Like extremely	15-cm non structured
CATA emojis	😀 😐 🤨 😋 🙄 🥴 😱 😊 😏 😍 🤮 😲 😕 😠 🥺	Check-All-That-Apply (CATA)
Face scale	🙁 – 😐 – 😃	15-cm non structured
Perceived Quality	Inacceptable – Extraordinary	15-cm non structured

Supplementary Table 2. Volatile compounds profile (RPA, %) identified by HS-SPME-GC/MS in six beer brands commercialized in Mexico.

Volatile compound	Aroma[1]	CAS number	TF-STU	TF-IPA	BF-PIL	BF-0AL	BF-MUN	BF-VNA
Fatty Acids								
Hexanoic acid	Fatty, Cheesy, Sour	142-62-1	1.471 ± 0.132 [a]	1.513 ± 0.048 [a]	0.625 ± 0.012 [b]	0.685 ± 0.038 [b]	0.592 ± 0.043 [b]	0.384 ± 0.004 [c]
Octanoic acid	Fatty, Cheesy, Soapy, Caprylic, Rancid	124-07-02	23.673 ± 0.343 [a]	15.879 ± 0.546 [c]	14.683 ± 1.040 [c]	20.706 ± 0.498 [b]	12.427 ± 0.352 [d]	9.965 ± 0.298 [e]
Decanoic acid	Rancid, Sour, Fatty	334-48-5	9.434 ± 0.593 [a]	2.439 ± 0.112 [c]	3.382 ± 0.479 [c]	6.149 ± 0.266 [b]	2.935 ± 0.175 [c]	8.313 ± 0.676 [a]
Fatty Acid Esters								
Ethyl hexanoate	Fruity, Pineapple, Green, Sweet	123-66-0	1.156 ± 0.044 [bc]	1.612 ± 0.043 [a]	1.241 ± 0.129 [b]	0.660 ± 0.019 [d]	1.147 ± 0.042 [bc]	0.977 ± 0.047 [c]
Ethyl octanoate	Apple, Banana, Pineapple	106-32-1	8.085 ± 0.573 [bc]	6.203 ± 0.672 [cd]	10.622 ± 1.269 [a]	0.487 ± 0.007 [e]	4.579 ± 0.212 [d]	8.762 ± 0.300 [ab]
Ethyl decanoate	Waxy, Apple, Grape, Sweet	110-38-3	12.721 ± 0.802 [a]	3.817 ±0.386 [cd]	4.941 ± 0.523 [c]	N.D.	2.509 ± 0.110 [d]	7.790 ± 0.149 [b]
Ethyl 9-decenoate	Fruity, Fatty	67233-91-4	0.333 ± 0.013 [c]	1.939 ±0.117 [a]	1.052 ± 0.120 [b]	0.261 ± 0.072 [c]	N.D.	0.983 ± 0.016 [b]
Ethyl laurate	Floral, Soapy, Sweet	106-33-2	2.860 ± 0.083 [bc]	2.281 ± 0.087 [c]	3.056 ± 0.132 [b]	0.199 ± 0.003 [d]	10.836 ± 0.476 [a]	3.490 ± 0.078 [b]
Ethyl myristate	Sweet, Waxy	124-06-01	0.312 ± 0.015	N.D.	N.D.	N.D.	N.D.	N.D.
Ethyl palmitate	Waxy, Fruity, Milky	628-97-7	0.862 ± 0.374	N.D.	N.D.	N.D.	N.D.	N.D.
Phenethyl palmitate	Sweet, Honey, Floral, Waxy, Woody	6290-37-5	N.D.	N.D.	N.D.	N.D.	N.D.	0.409 ± 0.042
Fusel Alcohols								
Isoamyl alcohol	Fusel, Alcoholic, Banana	123-51-3	21.531 ± 1.122 [cd]	25.711 ± 1.835 [b]	18.539 ± 0.885 [de]	17.586 ± 1.773 [e]	29.672 ± 0.754 [a]	24.373 ± 0.569 [bc]
2-Ethylhexanol	Citrus, Fresh, Oily	104-76-7	N.D.	N.D.	N.D.	N.D.	1.179 ± 0.026	N.D.
Phenylethyl Alcohol	Rose, Bread, Honey	60-12-8	10.436 ± 0.534 [d]	8.633 ± 0.523 [d]	19.815 ± 1.053 [bc]	27.057 ± 0.306 [a]	18.325 ± 0.771 [c]	21.291 ± 0.249 [b]
1-Decanol	Fatty, Waxy, Floral, Citrus	112-30-1	N.D.	N.D.	N.D.	N.D.	N.D.	0.385 ± 0.012
2-Undecanol	Fresh, Waxy, Cotton	1653-30-1	N.D.	1.601 ± 0.091	N.D.	N.D.	N.D.	N.D.
Fusel Alcohol Esters								
Isoamyl acetate	Sweet, Fruity, Banana,	123-92-2	1.317 ± 0.020 [d]	4.584 ± 0.238 [c]	8.294 ± 0.614 [b]	9.197 ± 0.143 [a]	7.660 ± 0.277 [b]	3.824 ± 0.138 [c]
Phenethyl acetate	Floral, Rose, Sweet	103-45-7	0.915 ± 0.056 [d]	1.590 ± 0.019 [d]	13.324 ± 0.766 [b]	15.470 ± 1.162 [a]	7.051 ± 0.103 [c]	6.691 ± 0.205 [c]
Texanol	Not reported	77-68-9	N.D.	N.D.	N.D.	0.501 ± 0.065 [a]	N.D.	0.476 ± 0.007 [a]
Isoamyl decanoate	Waxy, Banana, Fruity, Sweet, Cognac, Green	2306-91-4	0.547 ± 0.029	N.D.	N.D.	N.D.	N.D.	N.D.

Data indicate the mean of the RPA% (Relative peak area percent) present in each sample ± standard error (n=3). RPA% was defined as the ratio of the area of the component peak between the sum of all the areas of the identified peaks in the beer sample. Different letters denote significant differences between beers samples for each compound based on the Least Significant Difference (LSD, $p < 0.05$) method. Volatile compounds were identified by comparing the mass spectrum of the compound to the mass spectrum libraries of National Institute of Standards and Technology (NIST; National Institute of Standards and Technology, Gaithersburg, MD, United States), a match of >80% was considered for identification. N.D. indicate that the compound was not detected in the sample. [1]Aroma was reported based on the information of Praia et. al. (2022), Gonzalez Viejo (2019), The Good Scent Company (2018), and Liu and Quek (2016). Sample abbreviations were displayed in **Figure 1.**

Supplementary Table 2. Volatile compounds profile (RPA, %) identified by HS-SPME-GC/MS in six beer brands commercialized in Mexico (*Continuation*)

Volatile compound	Aroma[1]	CAS number	TF-STU	TF-IPA	BF-PIL	BF-0AL	BF-MUN	BF-VNA
Ketone								
2-Undecanone	Waxy, Fruity, Fatty, Floral	0112-12-9	N.D.	1.256 ± 0.023	N.D.	N.D.	N.D.	N.D.
Monoterpenoids								
Linalool	Citrus, Rose, Blueberry, Hops	78-70-6	0.610 ± 0.085 ^{b}	7.141 ± 0.511 ^{a}	N.D.	N.D.	N.D.	N.D.
Citronellol	Floral, Rose, Citrus	106-22-9	N.D.	1.439 ± 0.011	N.D.	N.D.	N.D.	N.D.
Geraniol	Sweet, Floral, Fruity, Rose, Citrus	106-24-1	N.D.	6.732 ± 0.052	N.D.	N.D.	N.D.	N.D.
Methyl geranate	Waxy, Green, Fruity	2349-14-6	N.D.	5.631 ± 0.610	N.D.	N.D.	N.D.	N.D.
Citronellyl acetate	Floral, Green, Rose, Citrus	150-84-5	N.D.	N.D.	N.D.	N.D.	N.D.	0.317 ± 0.002
Phenols								
2-Methoxy-4-vinylphenol	Spicy, Clove, Smoky	7786-61-0	N.D.	N.D.	N.D.	0.272 ± 0.010	N.D.	N.D.
Butylated hydroxytoluene	Mild phenolic camphor	128-37-0	1.026 ± 0.027	N.D.	N.D.	N.D.	N.D.	N.D.
2,4-Di-tert-butylphenol	Phenolic	96-76-4	N.D.	N.D.	N.D.	0.287 ± 0.011	N.D.	N.D.
Sesquiterpenoids								
Caryophyllene	Sweet, Woody, Clove	87-44-5	0.357 ± 0.004	N.D.	N.D.	N.D.	N.D.	N.D.
Humulene	Hops, Woody	6753-98-6	1.651 ± 0.022	N.D.	N.D.	N.D.	N.D.	N.D.
trans-Nerolidol	Floral, Green, Citrus, Woody, Waxy	40716-66-3	N.D.	N.D.	0.430 ± 0.035 ^{b}	0.483 ± 0.041 ^{ab}	0.418 ± 0.009 ^{b}	0.635 ± 0.080 ^{a}
Humulol	No reported	24405-58-1	0.279 ± 0.005	N.D.	N.D.	N.D.	N.D.	N.D.
tau-Cadinol	Balsam, Earthy	5937-11-1	0.427 ± 0.006 ^{a}	N.D.	N.D.	N.D.	N.D.	0.429 ± 0.011 ^{a}
alpha-Eudesmol	Woody, green	473-16-5	N.D.	N.D.	N.D.	N.D.	N.D.	0.508 ± 0.014
2,3-Dihydro farnesyl acetate	Floral, Green, Fruity	58130-58-8	N.D.	N.D.	N.D.	N.D.	0.192 ± 0.007	N.D.
Farnesyl acetate	Oily, Waxy	4128-17-0	N.D.	N.D.	N.D.	N.D.	0.481 ± 0.019	N.D.

Data indicate the mean of the RPA % (Relative peak area percent) present in each sample ± standard error (n=3). RPA% was defined as the ratio of the area of the component peak between the sum of all the areas of the identified peaks in the beer sample. Different letters denote significant differences between beers samples for each compound based on the Least Significant Difference (LSD, $p < 0.05$) method. Volatile compounds were identified by comparing the mass spectrum of the compound to the mass spectrum libraries of National Institute of Standards and Technology (NIST; National Institute of Standards and Technology, Gaithersburg, MD, United States), a match of >80% was considered for identification. N.D. indicate that the compound was not detected in the sample. [1]Aroma was reported based on the information of Praia et. al. (2022), Gonzalez Viejo (2019), The Good Scent Company (2018), and Liu and Quek (2016). Sample abbreviations were displayed in **Figure 1.**

Supplementary Figures

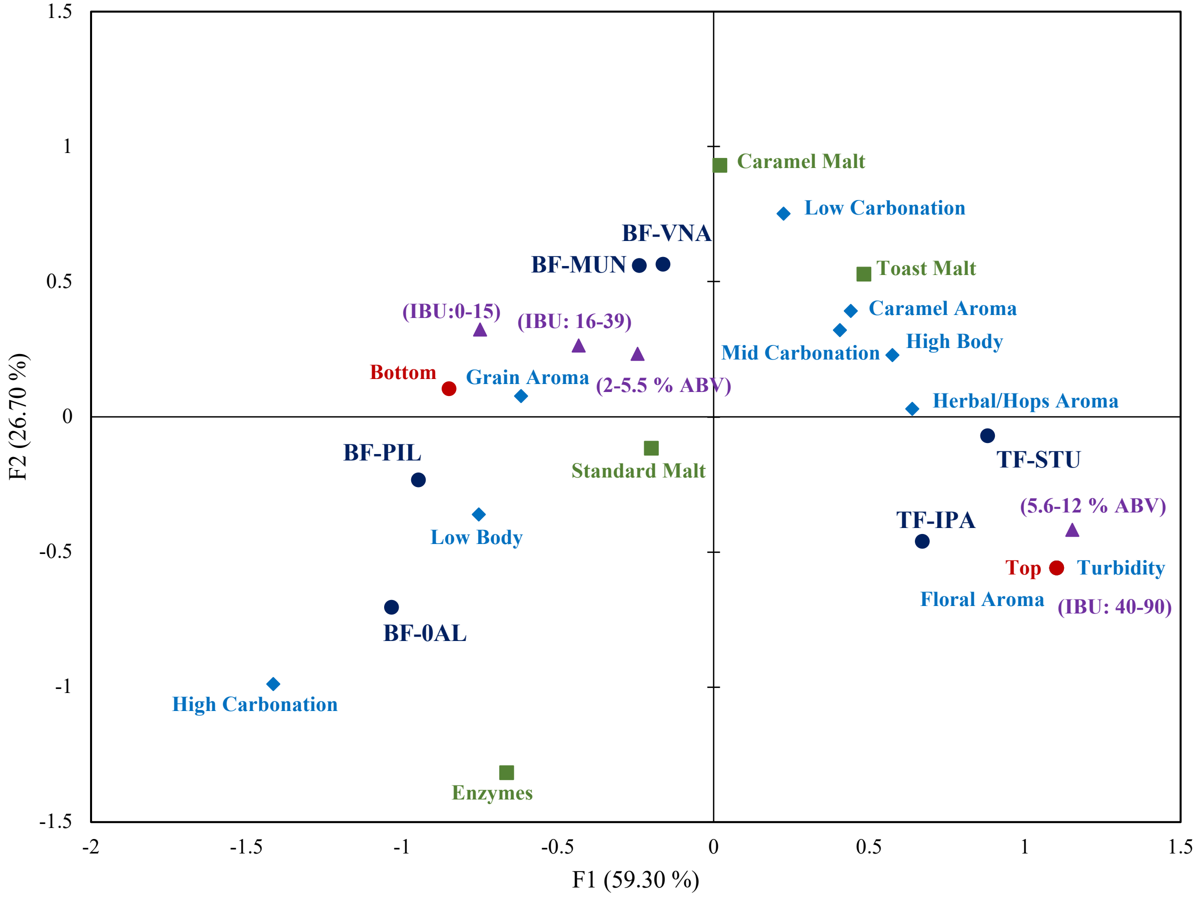

Supplementary Figure 1. Processing and composition characteristics of the commercial beer samples which defined their style according to industrial guidelines. ABV = alcohol by volume, IBU = International bitterness units. Samples information and abbreviations were defined in Figure 1 of the manuscript, and included BF-PIL, BF-0AL, BF-MUN, BF-VNA, TF-STU, and TF-IPA. ● Navy blue represents the sample identification abbreviations with its beer style, ■ Green represents the ingredients that are normally used to produce a beer, ◆ cerulean represents sensory traits that must be present in each beer style, ● red represents the type of fermentation, ▲ purple represents the physicochemical traits of each beer style. Beer style guidelines were obtained from Gatza *et al*., 2023 and Strong *et al*., 2021.

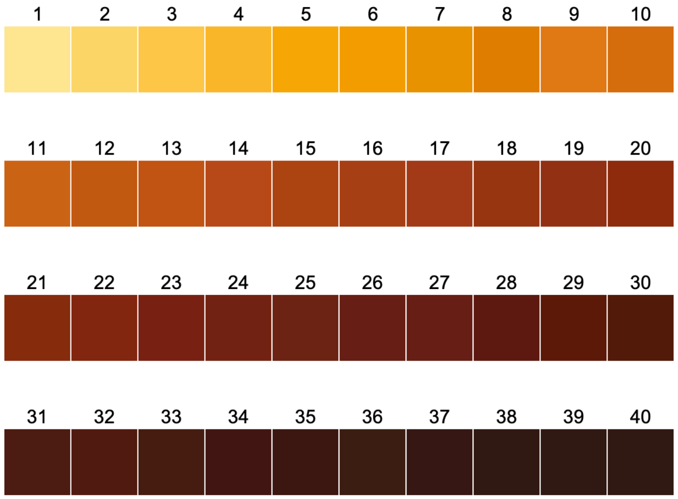

Supplementary Figure 2. Standard Reference Method (ºSRM) **color scale**. Figure generated with the hexadecimal color codes of Brewer's Friend (2022).

REFERENCES

Brewer'sFriend. (2022). *Beer Color Converter*. https://www.brewersfriend.com/color-calculator/

Gatza, P., Skypeck, C., Kirkpatrick, K., Williams, C., Parr, S, Rabin, D. (2023). Brewers Association. Beer Style Guidelines. Obtained from https://cdn.brewersassociation.org/wp-content/uploads/2023/07/10124402/2023_BA_Beer_Style_Guidelines-updated.pdf

Gonzalez Viejo, C., Fuentes, S., Torrico, D. D., Godbole, A., & Dunshea, F. R. (2019). Chemical characterization of aromas in beer and their effect on consumers liking. *Food Chemistry*, *293*. https://doi.org/10.1016/j.foodchem.2019.04.114

Liu, S. Q., & Quek, A. Y. H. (2016). Evaluation of beer fermentation with a novel yeast Williopsis saturnus. *Food Technology and Biotechnology*, *54*(4). https://doi.org/10.17113/ft b.54.04.16.4440

Praia, A. B., Herkenhoff, M. E., Broedel, O., Frohme, M., & Saad, S. M. I. (2022). Sour Beer with Lacticaseibacillus paracasei subsp. paracasei F19: Feasibility and Influence of Supplementation with Spondias mombin L. Juice and/or By-Product. *Foods*, *11*(24). https://doi.org/10.3390/foods11244068

Strong, G., & England, K. (2021). Beer judge certification program. Beer Style Guidelines. Obtained from https://www.bjcp.org/download/2021_Guidelines_Beer.pdf

The Good Scents Company. (2018). *Flavor, Food and Fragrance Industry*. http://www.thegoodscentscompany.com/search2.html
